# Shall the Charter of the New-York Medical Society Be Surrendered?

**Published:** 1847-01

**Authors:** 


*Shall the Charter of the New-York Medical Society be Surrendered?*—It appears that an effort has recently been made by some members of the N. Y. City Med. Society to surrender the charter of the Society. We quote the following remarks on the subject, by the editor of the N. Y. Journal of Medicine and the Collateral Sciences.

We have been unable to attend the different meetings of our County Medical Society, recently convened for the purpose of discussing the question whether its charter should be surrendered to the Legislature, the power from whence it was derived. We have, therefore, heard none of the arguments adduced on either side of the question, and the conclusion at which we have arrived is the result of our own unbiassed and deliberate judgment. We believe, then, that the Society should retain all its corporate rights and privileges, and wherein they are adequate for the attainment of the ends for which the Society was originally constituted, that it should petition the Legislature for such a modification or extension of its creation. The Society should certainly possess the power of admitting and expelling members according to its by-laws, without resorting to the judiciary; in other words, a right to judge of the qualifications of its members—a right which every other incorporated or voluntary society possesses, and without which no society can long exist. It should, in connection with the other County Societies, ask for a repeal of all those regulations, concerning the profession, which are inconsistent with such power; in short, that the societies shall have the power of regulating their affairs by such by-laws as each society shall, in its majority capacity, deem right and proper for its own interests. That the Legislature would listen to such a petition, there can be no reasonable doubt whatever entertained. We have heard of no disposition on the part of the other County Societies to relinquish their charters, and we know of no good reason why they should do so. The principal argument urged for the adoption of such a course by our own Society, we understand to be the want of disciplinary power. It is contended by many, however, that it already has such power, (see Sec. 13th of the "*Statutes*," and Sec. 3d, of the "*General Regulations*," and the *By-Laws*, Art. 1 and 5th; also the *Covenanting Obligation*, xv., 3.) But to place this matter beyond all doubt, we think a special act should be obtained from the Legislature for that purpose. The advantage possessed by a corporate body over a voluntary society, are too obvious to dwell upon here, and we should be sorry to see them voluntarily relinquished, without gaining an equivalent. That the Society, under its present organization, lacks the principle of vitality, we think, is sufficiently evident; and so far as we recollect, it has never been a very efficient laborer in the cause of science, even when it possessed the powers of which it is now supposed to be deprived. We should be rejoiced to see it arouse from its lethargy, and take that position in the medical world to which it is entitled from the number and respectability of its members; and all that is necessary to ensure this result is, that *each member should do his duty.*

